# Cortical movement of Bicoid in early *Drosophila* embryos is actin- and microtubule-dependent and disagrees with the SDD diffusion model

**DOI:** 10.1371/journal.pone.0185443

**Published:** 2017-10-03

**Authors:** Xiaoli Cai, Mira Akber, Alexander Spirov, Stefan Baumgartner

**Affiliations:** 1 Department of Experimental Medical Sciences, Lund University, Lund, Sweden; 2 Computer Science Department, Stony Brook University, Stony Brook, NY, United States of America and Sechenov Institute of Evolutionary Physiology and Biochemistry, St. Petersburg, Russia; Texas A&M International University, UNITED STATES

## Abstract

The Bicoid (Bcd) protein gradient in *Drosophila* serves as a paradigm for gradient formation in textbooks. The SDD model (synthesis, diffusion, degradation) was proposed to explain the formation of the gradient. The SDD model states that the *bcd* mRNA is located at the anterior pole of the embryo at all times and serves a source for translation of the Bicoid protein, coupled with diffusion and uniform degradation throughout the embryo. Recently, the ARTS model (active RNA transport, synthesis) challenged the SDD model. In this model, the mRNA is transported at the cortex along microtubules to form a mRNA gradient which serves as template for the production of Bcd, hence little Bcd movement is involved. To test the validity of the SDD model, we developed a sensitive assay to monitor the movement of Bcd during early nuclear cycles. We observed that Bcd moved along the cortex and not in a broad front towards the posterior as the SDD model would have predicted. We subjected embryos to hypoxia where the mRNA remained strictly located at the tip at all times, while the protein was allowed to move freely, thus conforming to an ideal experimental setup to test the SDD model. Unexpectedly, Bcd still moved along the cortex. Moreover, cortical Bcd movement was sparse, even under longer hypoxic conditions. Hypoxic embryos treated with drugs compromising microtubule and actin function affected Bcd cortical movement and stability. Vinblastine treatment allowed the simulation of an ideal SDD model whereby the protein moved throughout the embryo in a broad front. In unfertilized embryos, the Bcd protein followed the mRNA which itself was transported into the interior of the embryo utilizing a hitherto undiscovered microtubular network. Our data suggest that the Bcd gradient formation is probably more complex than previously anticipated.

## Introduction

The maternal *bicoid* (*bcd*) gene in *Drosophila* is described a paradigm in textbooks for gradient formation. To date, there are two prevailing models describing how the gradient is established in the first 3 hours of embryonic development. The first one, termed SDD model, after synthesis, diffusion and uniform degradation [1] states that the *bcd* mRNA stays at the anterior tip at all stages and that, upon translation of the protein, Bcd diffuses to the posterior, followed by uniform degradation. It is noted that diffusion has not been demonstrated experimentally, nor has it been shown in which path of the early embryo Bcd chooses to move to the posterior. In essence, the SDD model was proclaimed as a dogma without experimental evidence and has stayed in textbooks for more than two decades as a paradigm for gradient formation. The SDD model was challenged in 2009 with a report showing that the Bcd protein movement is not the cause for gradient formation, but rather the existence of a *bcd* mRNA gradient which serves as template for Bcd a gradient protein translation [2–5]. Later, in 2011, the SDD model was modified to the “extended SDD model” by [6] whereby a small mRNA gradient reaching about 80% egg length would contribute, but would not fully account for Bcd protein gradient formation, concluding that the major cause for gradient formation would still be Bcd protein movement.

To validate the diffusion model whose basic assumption had never been rigorously tested since its establishment in 1988 [1], fluorescent dextran particles were injected at the very tip of the embryo leading the author to conclude that the motion of the dextran particles fits the diffusion equation well [7]. Most importantly, the particles spread uniformly over the whole inner area leading to the final conclusion that the movement of Bcd would conform to the same diffusion properties as dextran particles.

Bcd protein distribution and posterior migration in early nuclear cycle (nc) 2–6 embryos was analyzed in detail by [8]. In addition to the posterior movement, a deep internal plume of Bcd was detected during nuclear cycles 4–6 enwrapping internal nuclei, thus recapitulating the expression of the *bcd* mRNA in a plume at nc 4 [3, 6, 8]. This observation led [8] to propose two models of Bcd gradient formation during early nuclear cycles. The first, termed “the continuous model”, would allow a continuous redistribution of both the mRNA and the protein entirely at the cortex, while the second one, termed “2-step model”, would imply that the *bcd* mRNA and protein would generate an interior plume during nc 4–6, followed by the generation of a second gradient at the cortex during the blastoderm stages.

Exposure of *Drosophila* to hypoxia has been described in detail [9–15]. The majority of the studies were performed on adult flies, while only a few reports dealt with embryogenesis [9, 10, 16]. The first marked signs of oxygen deprivation exerted at early nuclear stages was a reversible developmental arrest, with younger embryos being more sensitive and less able to resume development than older embryos [9, 16]. An immediate reaction of internal nuclei to hypoxia was often, but not always, a characteristic condensation and movement of the DNA to the inner surface of the swollen nuclei, giving them a typical ring-like structure [9], S2 Fig). Whether the nuclei adopted a ring-shaped configuration or not depended solely on whether nuclei arrest occurred at interphase or at metaphase where O_2_ deprivation was induced [10]. Another marked feature of hypoxic embryos was the fast recovery to normal development, occurring within ~ 10 minutes [9, 10]. All these above aspects allowed us to study Bcd protein movement in an experimental set-up ideal for testing the SDD model.

It is well known that drugs can influence *bcd* localization in oocytes and embryos. Most studies with drugs were performed with the intention to study the behavior of the *bcd* mRNA in oocytes [17, 18] and to a lesser extent also in the embryo [5]. The most commonly used drugs were those directed against the two major cytoskeletal components, the microtubules (MTs) and actin. To compromise the function of MTs, the MT-degrading drugs colchicine and colcemid were mostly used in the past [19]. If *Drosophila* oocytes were bathed in these drugs, the *bcd* mRNA did not localize properly to the anterior [18, 20, 21] which suggested that the MT-based transport of *bcd* mRNA was compromised. The drug vinblastine was shown to bind to a distinct site between Tubulin heterodimers [19], leading to the degradation of MTs, but was not used in *bcd* localization so far. Finally, taxol was described as a MT-stabilizing drug [19] used in the past to visualize MTs in early *Drosophila* embryos [22–25]. However, taxol elicited artefacts and thus did not reveal the true architecture of early cortical MTs, leading to alteration of the appearance of the anterior cortical network [5].

Actin was shown to be crucial for anterior *bcd* mRNA tethering at the end of oogenesis. If oocytes were incubated in the actin-depolymerizing drug cytochalasin D, *bcd* mRNA localization at the anterior end was compromised and stable actin-dependent anchoring of the mRNA was no longer possible [17, 20]. Likewise, the actin cytoskeleton was shown to be required for the maintenance of polar plasm components such as the *nanos* or *oskar* mRNA [26]. Other actin-targeting drugs exist [27, 28], such as. the latrunculins, an actin-destabilizing drug similar to cytochalasin D which was used in the past to disrupt filamentous actin (F-actin) in the early embryo [29]. Another is the F-actin-stabilizing drug phalloidin which is mostly used as a tool to visualize F-actin in combination with fluorescent phalloidin-derivatives.

To monitor Bcd movement in early nc embryos, we developed a sensitive assay, coupled with the ability to apply drugs that influence Bcd movement. We demonstrate that the Bcd protein migrates along the outermost cortex. Furthermore, Bcd migration is microtubule- and actin-dependent, suggesting that the Bcd gradient formation is probably more complex than previously anticipated.

## Material and methods

### Fly stocks

To ensure high levels of Bcd protein, the P (*bcd*^*+5+*8^) / FM7 stock was used (T301, Tübingen stock list, gift of Tom Kornberg). Unfertilized eggs in larger quantities were obtained from females transgenic for the male sex peptide, P[rye[+t7.2] = Acp70A[g.Yp1.hs]]G10 (Bloomington stock number 4365). In all cases, pre-collections were used to ensure correct age of the laid embryos. *bcd* mRNA patterns in those G10 embryos were indistinguishable from those of unfertilized wild-type embryos.

### Hypoxia and drug treatment

**Fig 1:**
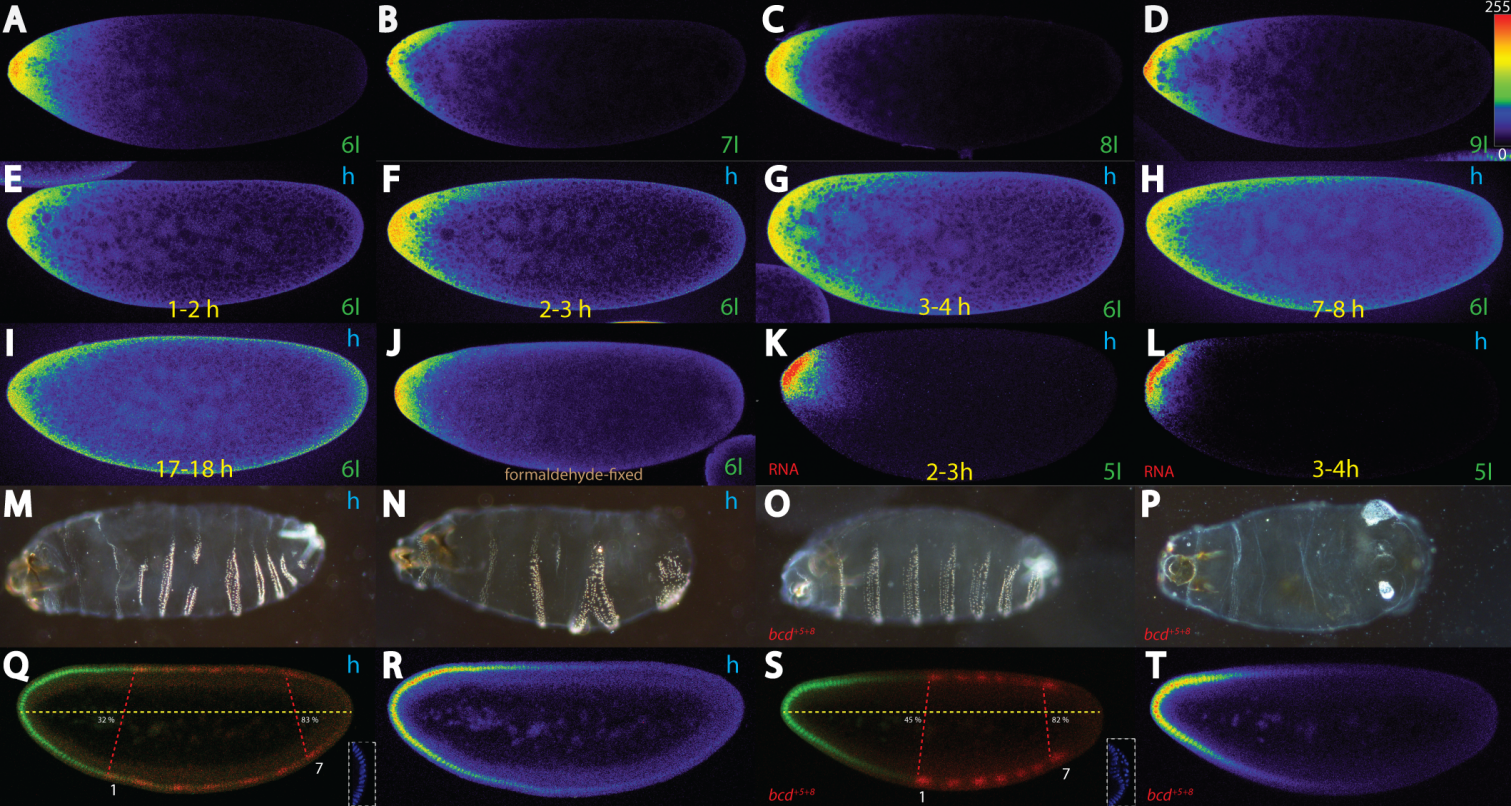
Cortical movement of Bcd in hypoxic embryos and effects of hypoxia on the segmental axis. Pictures represent midsagittal confocal planes of embryos oriented with their dorsal side up and anterior to the left, except for (M-P) which show cuticles. Relative intensities of the crude confocal pictures were converted to a color scale with values of 0–255 (8-bit), shown in (D), except for (M) to (Q) and (S). Nomenclature of nuclear cycles follows that of [46]. (A-D) untreated *bcd*^*+5+8*^ embryos stained with Bcd antibodies. (A) interphase of nuclear cycle (nc) 6, (B) nc 7, (C) nc 8, (D) nc 9 embryos. Note the migration of the protein at the cortex of the embryo and not to the interior. (E-I) Relative Bcd intensities of nc 6 embryos in hypoxic *bcd*^*+5+8*^ embryos and collected at different time intervals after hypoxia treatment: (E) 1–2 h, (F) 2–3 h, (G) 3–4 h, (H) 7–8 h, (I) 17–18 h. Note the movement of the Bcd protein in the “sleeping” embryos along the cortex. (J) Formaldehyde-fixed nc 6 embryo stained with Bcd antibodies. (K-L) distribution of the *bcd* RNA in *bcd*^*+5+8*^ embryos after 2–3 h hypoxia (K) and 3–4 h hypoxia (L). Note the sparse movement of the mRNA. Weak (M) and strong cuticle phenotype (N) of wild-type (Oregon R) embryos collected in a 1 hour interval, subjected to 3 h hypoxia and recovered for 36 h. Weak (O) and strong cuticle phenotype (P) of *bcd*^*+5+8*^ embryos. (Q) 0–1 h collected, 3 h hypoxia-treated and 3 h recovery nc 14 embryo, stained for Bcd protein (green) and Eve (red), percentages indicate position of Eve stripes 1 and 7 in % egg length, respectively. Insert in (Q) shows DAPI staining of the posterior end demonstrating lack of pole cells. (R) color conversion of the Bcd pattern in (Q). (S) nc 14 *bcd*^*+5+8*^ embryo stained for Bcd protein (green) and Eve (red). Percentages indicate position of Eve stripes 1 and 7 in % egg length, respectively. (T) color conversion of the Bcd pattern in (S).

Embryos were collected at 25° (using precollection) in 1 hour intervals and were exposed to hypoxia with or without drug treatment for the time indicated. The assay was as follows: After dechorionation and rinsing with tap water, embryos were transferred to a cap which was cut off from an Eppendorf vial and filled with 200 μl PBT, supplemented by 1/100 volume of embryo permeabilization buffer [52]. The solution was evacuated with a water vacuum pump to remove any oxygen, and drugs were added to the final concentration: 50 μg/ml colchicine / 20 μg/ml colcemid mixture, 10 μM taxol, 10 μM vinblastine, 20 μg/ml latrunculin B and 20 μg/ml phalloidin. Embryos were transferred to the cap using a brush, aided by a fine forceps to remove embryos from the brush tip. All embryos sank down to the bottom despite the fact that they still harbored the vitelline membrane. Embryos were incubated for the indicate time interval in a moisture chamber at 25°C. Addition of heptane was strictly avoided as it led to a substantial increase of background during immunofluorescence. Embryos were fixed using either 4% formaldehyde (for *in situ* hybridization and Bcd antibody staining in Fig 1J), or using heat-fixation (for all other embryos). Molecular markers in S2 Fig were Hoechst 33342, used at 50μg/ml, Sytox Green and TO-PRO3 (Molecular Probes), used at 20 μg/ml, respectively.

### Antibodies and image recording

Rabbit antibodies against full-length Bcd were a gift from M. Biggin and were used at 1:500. Goat-anti Staufen antibodies (Santa Cruz Biotechnology) (used in S1 Fig) were used at 1:20. Rat antibodies against Staufen were a gift from A. Ephrussi and were used at 1:1000. mab JLA20 directed against actin (DHSB) was used at 1:50. All confocal pictures were recorded on a Zeiss LSM 710 at 8-bit resolution allowing 256 intensity values. Each embryo was recorded as a stack of 6–10 pictures from a start point before the middle of the embryo to an end point beyond the middle which then served as the basis to decide upon the mid-sagittal-most section. Care was taken to ensue that the gain of all recordings was adjusted to avoid saturation of the peak intensities by adjusting the gain of the LSM 710. For color conversion and interpretation of signal intensities, the OsiriX DICOM program was used [33].

### Data acquisition

**Fig 2:**
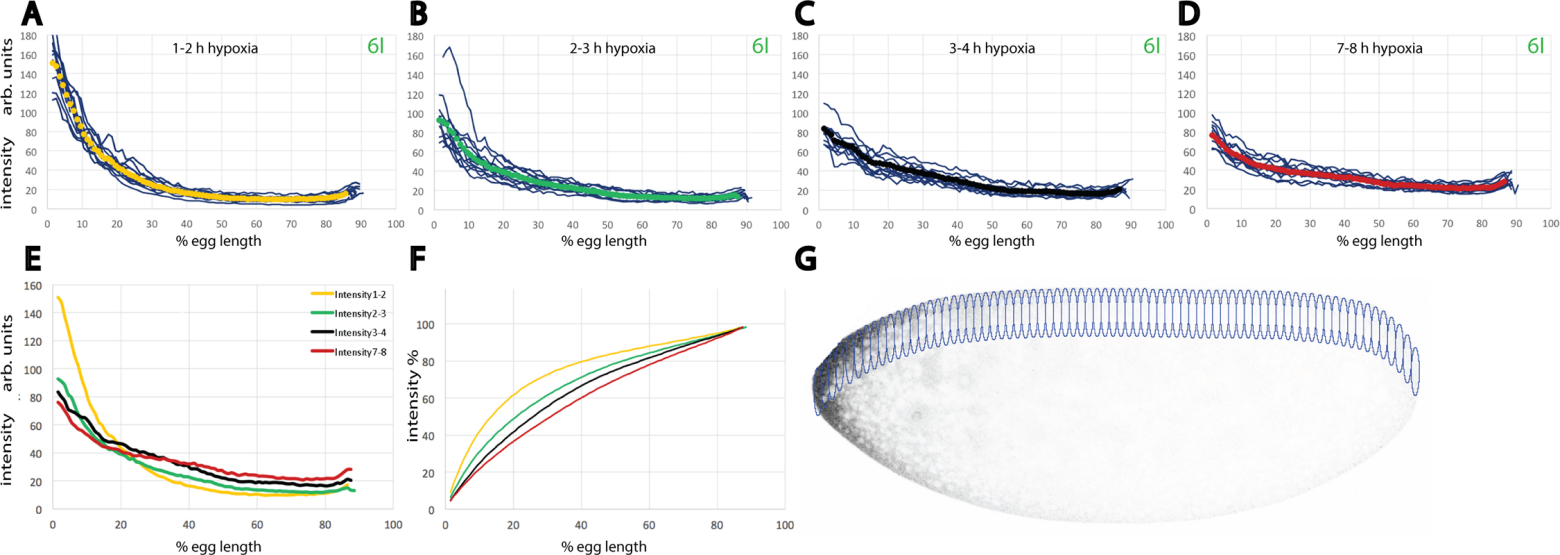
Batch analysis of hypoxic embryos demonstrates that the extent of Bcd movement changes with time. (A-D) Bcd protein profiles of embryos at nc 6 exposed to hypoxia. (A) 12 embryos exposed to hypoxia for 1 h and shown along with a yellow line as mean profile, (B) 12 embryos exposed to hypoxia for 2 h and shown along with a green line as mean profile, (C) 12 embryos exposed to hypoxia for 3 h and shown along with a black line as mean profile, (D) 12 embryos exposed to hypoxia for 6 h and shown along with a red line as mean profile. Profiles were measured as illustrated in Fig 2G and shown as intensities with arbitrary units. (E) Superimposition of all 4 mean profiles from (A-D), shown as intensities with arbitrary units. (F) plots of (E) shown as percentage of the total Bcd protein content at a given point of the A-P axis. (G) Overview of an embryo, illustrating positions at which Bcd protein levels were measured in overlapping ellipsoid discs along the dorsal cortex of embryos.

Intensity graphs (Fig 2) were obtained from a ellipsoid area moved along the dorsal cortex of midsagittal sections, as illustrated in Fig 2G [53]. A detailed description of used algorithms, scripts, and tools is available on request.

### *In situ* hybridization

Fluorescent *in situ* hybridization (FISH) was used according to [3], except that RNA probes were used, combined with a home-made Alexa Fluor 555 Signal-Amplification Kit, using identical steps as the commercially-available Alexa Fluor 568 Signal-Amplification Kit (Thermo Fisher A11066).

## Results

### Bcd moves at the cortex and not to the interior during early nuclear cycles

To analyze Bcd movement during early nuclear cycles combined with the ability to use drugs that perturb Bcd migration, we tested numerous fixation and antibody staining protocols that would allow the combination of both approaches with an adequate signal-to-noise ratio. Since the formaldehyde fixation conditions as described in [8] constantly led to unwanted noise in combination with hypoxia and drug treatment (see below), we used heat-fixation instead. This fixation method was shown to work reliably in the past for Bcd antibody staining [3, 30]. To increase the sensitivity, we utilized a strain which produces 3 times more Bcd protein than wild-type, *bcd*^*+5+8*^ [8, 31, 32]. Moreover, the increased levels of Bcd permitted the analysis of single confocal midsagittal sections that allowed a precise analsyis of the spatial pattern of Bcd.

*bcd*^*+5+8*^ embryos are known to generate a distinct Bcd gradient which differs from that of wild-type embryos [1]. We monitored the distribution of Bcd in *bcd*^*+5+8*^ embryos in early cleavage staged embryos and used single confocal pictures derived from midsagittal stacks. To monitor the relative distribution without the necessity to graph the intensities along the A-P axis, we converted the intensities from the crude confocal file to a color scale [3, 33] allowing us to monitor the spatial distribution of Bcd. *bcd*^*+5+8*^ embryos produce about three times more Bcd protein than wild-type embryos (S1A, S1B, S1D and S1E Fig), but the appearance of the initial gradient of Bcd in *bcd*^*+5+8*^ embryos is retarded at nc 6 compared to an identical nc 6 wild-type embryo. This was evident from comparing the heat maps of the respective caps (S1D and S1E Fig). This observation suggests that the system for transporting the mRNA is not scaled up to accommodate for the transport of the high amount of *bcd* mRNA in *bcd*^*+5+8*^ embryos. In a nc 6 embryo (Fig 1A), Bcd is strongly concentrated at the tip with some posterior migration on the dorsal and ventral side, recapitulating the distribution of the mRNA, also confirmed in [6, 8] at nc 4. At nc 7, however, this distribution changes dramatically and the protein covers an anterior cap with little Bcd in the inside (Fig 1B) and a smaller gradient extending to about 15% of the egg length. During the next two nuclear cycles (Fig 1C and 1D), the cap with the gradient expands to the posterior reaching about 25% at nc 9 (Fig 1D). To rule out that the heat-fixation procedure would extract Bcd protein such that only a subset of Bcd was revealed, we stained a formaldehyde-fixed nc 6 embryo (Fig 1J) that showed a staining pattern comparable to that of a heat-fixed nc 6 embryo (Fig 1A).

### Hypoxia and uptake of smaller molecules

To further investigate the movement and the path of the Bcd protein along the cortex, we decided to inactivate the mRNA transportation system without affecting the viability of the embryo. We had previously shown that the *bcd* mRNA is transported along cortical MTs in the early embryo [5]. Hence, it would be desirable to selectively inactivate cortical MTs without affecting the interior MT network involved in nuclear division and migration, a technically-challenging scenario, as any disturbance of the function of MTs has deleterious effects on the viability of the embryo.

To overcome the above constraints, we noted that embryos exposed to hypoxia arrest growth and are virtually “sleeping”, but are readily reactivated once oxygen is supplied [9, 10]. To this end, we developed an assay to submerge embryos into water-based buffer systems rather than expose them to argon as in other studies [9, 10]. Our approach also allowed drugs to move into the embryo. Other drug deliver assays into embryos have been previously described [34, 35] using detergents to help to permeabilize the embryo. The disadvantage was that many of the embryos suffered from the treatment. In contrast, our simple assay allowed for the simultaneous application of any drug in the hypoxic state, despite the presence of the water-repelling wax layer and the robust vitelline membrane [36].

To illustrate movement of smaller substances through the membranes, we assayed embryos under hypoxia by adding fluorescent substances up to a molecular weight of 1500 (S2 Fig). During hypoxia for 2 hours and simultaneous incubation with nuclear staining tools coupled to fluorochromes, we were able to detect fluorescence from the DNA stain Hoechst 33342 (S2A and S2E Fig), the DNA marker Sytox Green (S2B and S2F Fig) and the DNA marker TO-PRO3 (S2C and S2G Fig), all associated with the ring-type chromatin (S2D and S2H Fig). In many cases, strong fluorescence was observed at the anterior end (data not shown), suggesting that the most likely point of entry is at the micropyle, the location where the sperm enters the egg.

### Bicoid movement without mRNA gradient still occurs at the cortex

We reasoned that the sleeping period would allow us to monitor the movement of the Bcd protein and to assess how fast and how far the Bcd protein can move in the time where gradient formation was predicted to occur.

We chose the 6^th^ nuclear cycle, i. e. embryos with 32 nuclei as a reference time point to evaluate Bcd protein migration from the position where hypoxia was applied. To this end, *bcd*^*+5+8*^ embryos were collected during 1 hour intervals, incubated under hypoxic conditions and fixed. Only embryos at the 6^th^ nuclear division were recorded. In 1–2 h embryos (1 hour collecting, 1 hour hypoxia, Fig 1E), little movement of the protein was observed, compared to an untreated embryo (Fig 1A). In 2–3 h (2 hours-hypoxic) embryos (Fig 1F), the protein migrated to about 30% egg length (EL), but still the majority of the protein remained at the tip. In 3 hours-hypoxic embryos (Fig 1G), movement continued to about 50% EL, but the bulk was still detected at the tip. In 7 hours-hypoxic embryos (Fig 1H), Bcd protein has reached about 70% EL, revealing a flat gradient. After 17 hours of induced hypoxia, some Bcd protein has reached the posterior end, barely showing a gradient. Under these conditions, we can conclude one important finding: Bicoid protein does not move throughout the whole embryo, but rather follows a discrete path along the outmost part of the embryonic cortex, as it does under normal conditions (Fig 1A–1D).

To investigate if the *bcd* mRNA is the cause for the Bcd protein movement, we stained *bcd*^*+5+8*^ embryos that were exposed to hypoxia for the presence of *bcd* mRNA. To account for the time that is needed to synthesize Bcd protein, about 2 minutes [3, 37], we chose hypoxic nc 5 embryos, instead. In 2–3 h hypoxic embryos (Fig 1K), *bcd* mRNA was tightly located to the tip and very little movement was seen, consistent with its expression in wild-type embryos at a similar stage including the plume of interior mRNA [3, 6, 8]. In a 3–4 h hypoxic embryo (Fig 1L), little change was observed and the mRNA was still located at the tip. From these two time points, we can conclude, that 1) oxygen deprivation has an impact on the localization of the mRNA, i. e. it does not move in comparison with wild-type embryos, and 2) the mRNA is not the cause for the protein movement seen in Fig 1E–1I.

### Developmental consequences of hypoxia on segmental anlagen

To investigate the developmental consequences of hypoxia and the impact of Bicoid movement, we subjected wild-type Oregon-R embryos to 3 h hypoxia and allowed recovery for 36 hours in order to analyze the developmental consequences based on the cuticular pattern. The majority of the embryos (71%, S1 Table) showed the A-P axis affected, with an enlargement of the anterior segments and compression of the posterior segments, including head defects with shortened mouth hooks (Fig 1M). A lower portion of the embryos (24%, S1 Table) showed more severe A-P axis defects with several segments missing (Fig 1N). To compare the above effects to embryos exposed to high levels of Bcd, we monitored the cuticles of *bcd*^*+5+8*^ larvae. Since *bcd*^*+5+8*^ is a living stock, the majority (58%) of the offspring survive without any noticeable effect on A-P axis, also noted by [32]. The remaining 42% can be divided into mild defects (39%, S1 Table), where 2 thoracic segments, T2 and T3 were lacking, associated with defects in head-involution (Fig 1O). The remaining fraction (3%, S1 Table) contained embryos which showed a severely-affected A-P axis (Fig 1P) revealing no abdominal segments at all, while the head part was only mildly affected, revealing defects in head involution only. This data is consistent with results from *6x bcd* embryos [32]. To corroborate the cuticle defects caused by hypoxia, we stained embryos that were exposed to hypoxia for 3 hours followed by recovery for another 3 hours, with Bcd and Eve antibodies. While Bcd staining at first sight revealed a rather normal-looking gradient (Fig 1Q and 1R), Eve staining showed that all bands appeared stretched to the posterior (Fig 1Q), starting from 32% for stripe 1 to 83% to stripe 7, while in untreated wild-type embryos the stripes appeared from 32% to 75% [38–40]. Most conspicuously, however, no pole cells were observed (Fig 1Q, insert), suggesting that the fraction of posteriorly-migrated Bcd within the extra 3 hours was sufficient to suppress pole cell determination, indicative of altered fate of posterior nuclei. In comparison, the Bcd and Eve pattern in *bcd*^*+5+8*^ embryos showed a steep Bcd gradient (Fig 1S and 1T) while Eve stripes appeared compressed, starting from 45% to 82% (Fig 1S). In contrast to the hypoxic embryos, however, *bcd*^*+5+8*^ embryos still revealed pole cells (Fig 1S, insert). We can conclude that the migration of Bcd protein during 3 hours of hypoxia causes posterior defects not associated with *bcd*^*+5+8*^ embryos. Possibly, a distinct fraction of Bcd molecules may be transported to the posterior leading to suppression of pole cell formation.

### Quantitative analysis of cortical Bcd movement

To visualize cortical movement more precisely, we used the crude confocal data from larger batches of midsagittal sections from the time intervals as seen in Fig 1E–1L and analyzed the intensities by sliding an ellipsoid area along the dorsal side (Fig 2G). In contrast to the pictures in Fig 1 where the gain of the confocal microscope was individually adjusted to avoid saturation, the embryos of the batch series of Fig 2 were processed, collected and stained in the same experiment, and 12 nc 6 embryos from each hypoxic series were recorded in a single confocal session using identical intensity condition adjusted to the strongest signal of 1–2 h embryos. In 1–2 h hypoxic embryos (Fig 2A), the effect of extra Bcd movement appears minimal and the gradient looks similar to that of untreated *bcd*^*+5+8*^ embryos [1]. In 2–3 h hypoxic embryos (Fig 2B), however, a portion of the anteriorly-located Bcd protein has moved posteriorly, making the plot markedly flatter. This tendency continues in 3–4 h hypoxic embryos (Fig 2C). In 7–8 h hypoxic embryos (Fig 2D), the change of the shape of the plot is remarkably little compared to that of 3–4 h embryos (Fig 2C) suggesting that Bcd movement has come to a halt. Possibly, the embryo suffers from the prolonged hypoxia, associated with a high degree of lethality which is inherent to young embryos [9]. When the mean curves of Fig 2A–2D were compared (Fig 2E), 1–2 h hypoxic embryos (yellow) revealed a distinct curve compared to the remaining 3 curves whose slopes decreased the longer the embryos were exposed to hypoxia. This observation prompted us to monitor the percentaged distribution of Bcd in relation to the A-P axis after the different hypoxic incubations (Fig 2F). After 1–2 h hypoxia, still about 80% of Bcd was contained within the first 40.5% of the embryos. A marked change took place in 2–3 h embryos where 80% of the protein was contained within the first 52.5% of the embryo, representing a change of 12% during one hour. In 3–4 h embryos, the value was at 57.5% indicating a reduced movement in comparison to the previous interval. Most notably, during the following 3 hours (i. e. in 7–8 h embryos), only another 5% of Bcd moved to the posterior. From this experiment, we can conclude that Bcd movement was strongest during the early phases of hypoxia, but decreased substantially during the subsequent time intervals.

### Cortical movement of Bcd is microtubule-dependent

To investigate whether Bcd movement is dependent on cytoarchitectural changes of the egg, we combined our water-based hypoxia assay with drug application. To this end, we added the drugs directly to the buffer for the hypoxia-treated embryos and exposed them for the time interval indicated.

[5] exposed early nc embryos to the MT-destabilizing drug mixture colchicine/colcemid (CC) and could demonstrate that the anterior cortical MT network implicated in *bcd* mRNA transport was degraded. This data showed that drugs could enter the egg despite the water-repelling wax layer and the vitelline membrane, and that the MT network responded to the application.

**Fig 3:**
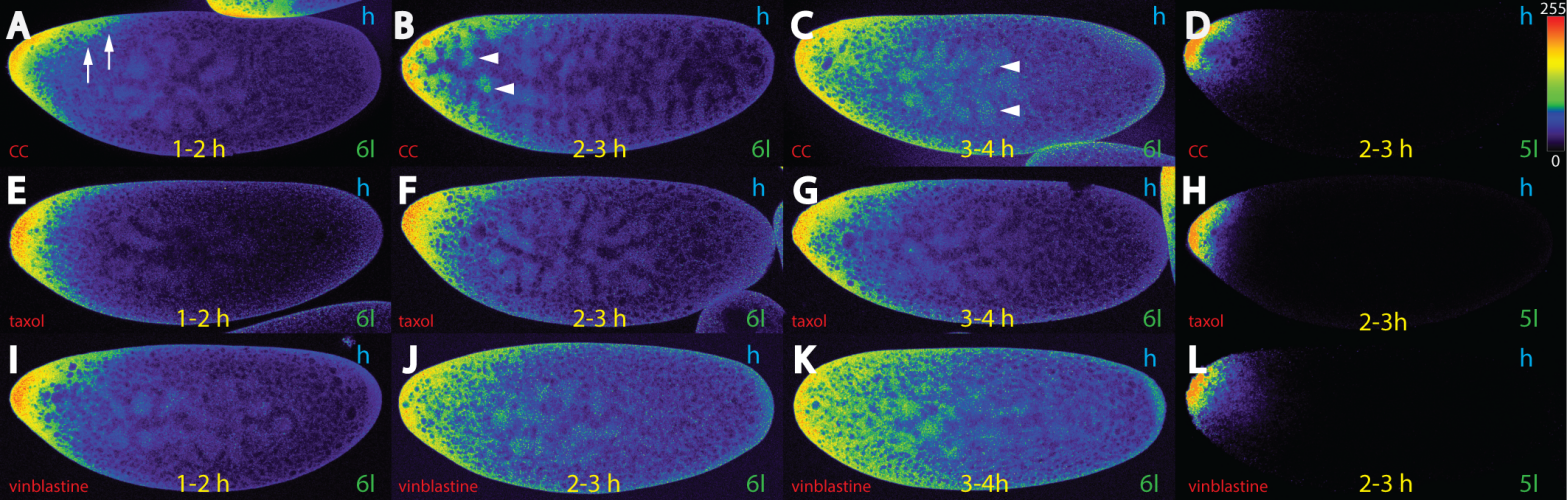
Bcd movement in hypoxia-treated embryos depends on microtubules. Pictures represent midsagittal confocal planes of embryos oriented with their dorsal side up and anterior to the left. Relative intensities of the crude confocal pictures were converted to a color scale with values of 0–255 (8-bit), shown in (D). (A-C) nc 6 *bcd*^*+5+8*^ embryos treated with colchicine/colcemid (“CC”) for 1–2 h (A), 2–3 h (B) and 3–4 h (C), and stained for Bcd. (D) nc 5 *bcd*^*+5+8*^ embryo treated with CC for 2–3 h, and stained for the *bcd* RNA. (E-G) nc 6 *bcd*^*+5+8*^ embryos treated with taxol for 1–2 h (E), 2–3 h (F) and 3–4 h (G), and stained for Bcd. (H) nc 5 *bcd*^*+5+8*^ embryo treated with taxol for 2–3 h, and stained for the *bcd* RNA. (I-K) nc 6 *bcd*^*+5+8*^ embryos treated with vinblastine for 1–2 h (I), 2–3 h (J) and 3–4 h (K), and stained for Bcd. (L) nc 5 *bcd*^*+5+8*^ embryo treated with vinblastine for 2–3 h, and stained for the *bcd* RNA. Note that destabilization of microtubules compromises the cortical path in favor of a more ubiquitous path. Note that the movement in (K) corresponds to the one predicted by the ideal SDD model where the protein will move in a broad front throughout the entire embryo.

Embryos exposed for 1 hour to hypoxia and CC exhibited subtle changes to the Bcd distribution pattern (Fig 3A), compared to untreated embryos (Fig 1E). Some protein moved more posteriorly (Fig 3A, arrows). If treated for 2–3 hours (Fig 3B), marked changes were observed. Parts of the protein migrated to the interior of the egg and some protein entered the energids, an actin-rich area surrounding the nuclei [24], as well as the nuclei (Fig 3B, arrowheads). Of note, in CC-treated hypoxic embryos, the chromatin never formed the typical rings (S2 Fig). In embryos treated for 3–4 hours (Fig 3C), the situation was even more pronounced with more Bcd migrating to the interior. Some Bcd even reached the nuclei in the middle of the embryo (Fig 3C, arrowheads). To exclude the possibility that the mRNA was the cause for this movement, we stained 2–3 hours-treated embryos for the presence of *bcd* mRNA (Fig 3D) which exhibited a distribution similar to that of hypoxia-only treated embryos (Fig 1K). This data demonstrated that Bcd movement was dependent on an intact MT network.

Hypoxia combined with taxol treatment revealed subtle changes in Bcd movement when exposed for 1–2 hours (Fig 3E) compared to the reference without drugs (Fig 1E). In 2–3 hours-treated embryos (Fig 3F), there was little change observed except that the protein as a bulk moved to the interior (Fig 3F). In 3–4 hours-treated embryos (Fig 3G), some posterior Bcd movement was observed, comparable to the reference (Fig 1G). If assayed for *bcd* mRNA distribution, taxol-treated embryos were almost indistinguishable from the reference (Fig 1K). We can conclude that taxol did not have a deleterious effect on Bcd movement, nor did it promote it substantially.

### Vinblastine treatment allows to simulate the SDD model

Vinblastine has been shown to affect MT growth, but has been shown to bind to a distinct site between heterodimers which are different to those by colchicine/colcemid and taxol (Florian and Mitchison, 2016). When vinblastine was applied during a 1–2 hour interval (Fig 3I), protein movement began in all directions, and became more obvious during the 2–3 h interval (Fig 3J). Here, the protein distributed equally over the whole inner part. In 3–4 hours-treated embryos (Fig 3K), the situation was even more pronounced. Protein distribution moved to the posterior in a broad front, and thus seemingly conforming to the SDD model. This data allows two interpretations: 1) Bcd protein movement requires an intact MT network in the yolk to prevent Bcd movement to the interior, 2) the cortical MT network no longer restricts Bcd protein movement to the cortex. To rule out that the cause for this massive migration being attributed to the mRNA, vinblastine-treated embryos were stained for the *bcd* mRNA revealing that *bcd* mRNA remained at the tip (Fig 3L), as obserevd in CC- or taxol-treated embryos (Fig 3D and 3H).

### Actin is indispensable for Bcd stability and cortical movement

**Fig 4:**
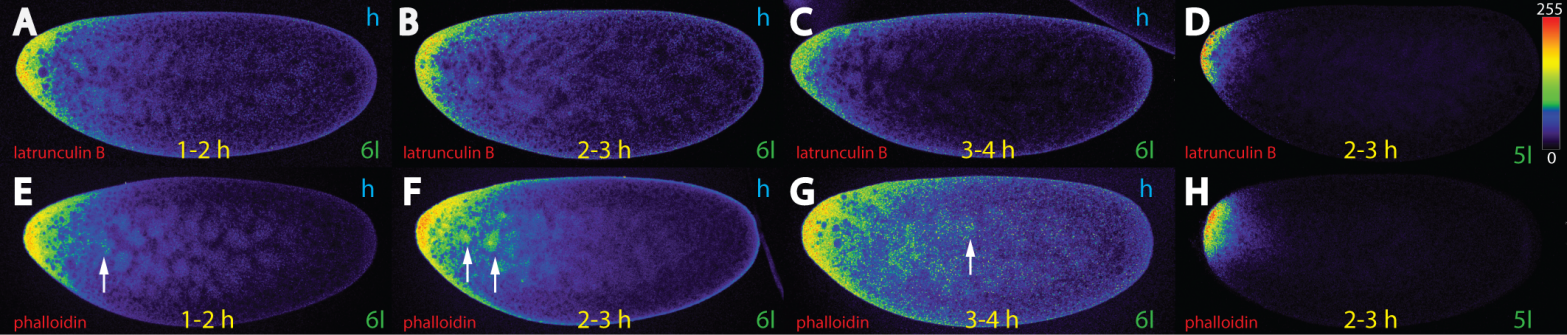
Bcd movement and stability in hypoxia-treated embryos depends on actin. Pictures represent midsagittal confocal planes of embryos oriented with their dorsal side up and anterior to the left. Relative intensities of the crude confocal pictures were converted to a color scale with values of 0–255 (8-bit), shown in (D). (A-C) nc 6 *bcd*^*+5+8*^ embryos treated with latrunculin B for 1–2 h (A), 2–3 h (B), 3–4 h (C) and stained for Bcd. (D) nc 5 embryo treated for 2–3 h with latrunculin B and stained for the *bcd* mRNA. Note that the Bcd protein does not move anymore along the cortex. (E-G) nc 6 *bcd*^*+5+8*^ embryos treated with phalloidin for 1–2 h (E), 2–3 h (F) and 3–4 h (G) and stained for Bcd. (H) nc 5 embryo treated for 2–3 h with phalloidin and stained for the *bcd* mRNA. Arrows in (E-G) denote interior nuclei, as well as energids revealing accumulation of Bcd protein. Note the decreased stability of Bcd in latrunculin B-treated embryos (A-C).

To investigate whether actin is involved in the movement, we subjected *bcd*^*+5+8*^ embryos to drugs such as phalloidin that prevents F-actin degradation (S3A and S3B Fig), or to latrunculin B that disrupts the formation of actin bundles (S3C and S3D Fig). In phalloidin-treated embryos, long cortical microfilaments (MFs) were observed (S3B Fig), consistent with a stabilization of F-actin [41]. In contrast, latrunculin B-treated embryos revealed absence of long MFs, and only globular actin particles were detected, instead (S3D Fig), demonstrating that these drugs can efficiently alter the actin cytoarchitecture. In 1–2 hours latrunculin B-treated-embryos (Fig 4A), initial movement of Bcd was indistinguishable from the reference (Fig 1E), while in 2–3 hours-treated embryos, sparse cortical movement and fewer Bcd protein particles were observed (Fig 4B), in comparison to the reference (Fig 1F). In 3–4 hours-treated embryos, no further movement occurred, but considerably lower amount of Bcd protein was observed, suggesting that the degradation of Bcd has commenced (Fig 4C). As far the mRNA was concerned, *bcd*^*+5+8*^ embryos treated for 2–3 h with latrunculin B did not show any mRNA movement nor were fewer mRNA molecules detected (Fig 4D), in comparison to the reference (Fig 1K). This suggests that the movement and the stability of Bcd protein was not dependent on the status of the mRNA. This data proposes that an intact actin network at the cortex is critically important for both Bcd movement and stability.

When the existing actin filaments were prevented from degradation, i. e. after exposure to phalloidin, we noted that Bcd movement initially (at the 1–2 hours interval) behaved like in a wild-type embryo, with the exception of a small fraction of the protein moving to the interior (Fig 4E, arrow). In 2–3 hours-exposed embryos, this behavior continued, resulting in Bcd staining in energids and interior nuclei (Fig 4F, arrows). Concurrently, a greater proportion of the Bcd protein followed the cortical pathway similar to control hypoxic embryos (Fig 1F). Longer exposure (3–4 hours interval) revealed further streaming of Bcd to the interior, and nuclei and energids showed staining with Bcd (Fig 4G, arrow), similar to embryos treated with CC (Fig 3C). However, it was not as extensive as that seen in vinblastine-treated embryos (Fig 3K). Again, the *bcd* mRNA did not move from the tip (Fig 4H), suggesting that cortical actin does not impede movement of Bcd.

### Bcd movement in unfertilized embryos is strictly dependent on the *bcd* mRNA which utilizes a MT pathway into the interior

**Fig 5:**
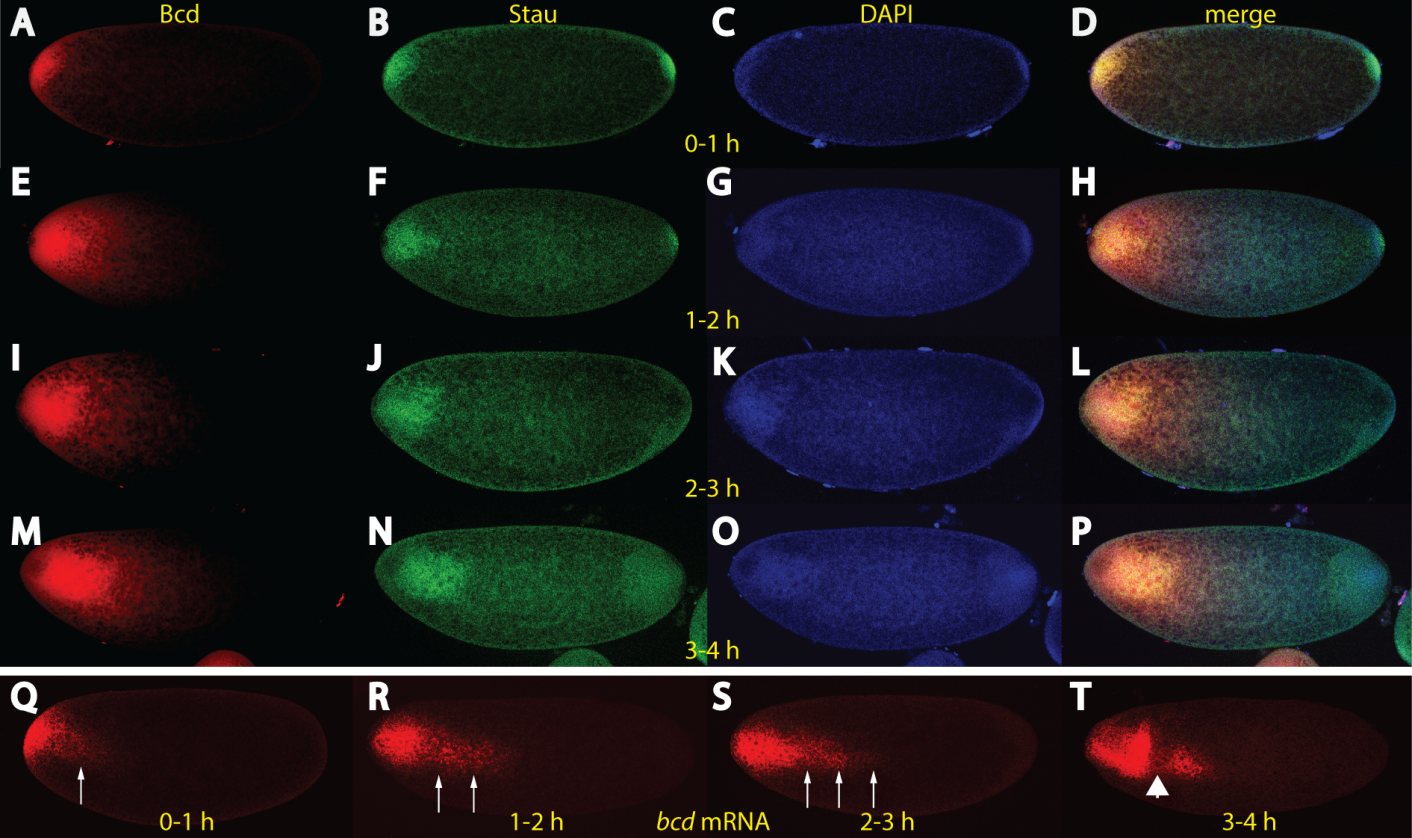
In unfertilized embryos, *bcd* mRNA and Bcd protein move to the interior. Unfertilized eggs collected from different time intervals, 0–1 h (A-D), 1–2 h (E-H), 2–3 h (I-L), 3–4 h (M-P), stained for Bcd protein (A, E, I, M), Staufen protein (B, F, J, N) and DAPI (C, G, K, O). The merge of all staining patterns is revealed in (D, H, L, P). Note the weak diffusion of the Bcd protein away from the bulk (A, E, I, M), which is always congruent to the Staufen protein (B, F, J, N). (Q-T*) in situ* hybridization of *bcd* mRNA of 0–1 h (Q), 1–2 h (R), 2–3 h (S) and 3–4 h (T) old unfertilized embryos. Arrows in (Q, R, S) denote fast movement of a portion of mRNA particles, apparently not associated with Staufen (compare to B, F J). Time intervals and proteins are indicated in yellow.

It is well documented that unfertilized eggs synthesize Bcd protein and that the process of fertilization is not a prerequisite for the initiation of Bcd translation [1, 8]. A recent study monitored Bcd movement in unfertilized embryos [42] showing that the Bcd gradient appeared longer and less steep compared to fertilized embryos of the same stage. The data was then interpreted in support of the SDD model. We therefore analyzed the patterns of Bcd and Staufen protein (as a read-out system for the *bcd* mRNA) in unfertilized embryos during short time intervals to monitor their movement. During the first hour, there was little diffusion of Bcd (Fig 5A) away from the source of the *bcd* mRNA template (Fig 5B). During the next hour (hours 1–2), Bcd showed expansion towards the posterior, with the bulk of protein associated in a region slightly shifted posterior from the tip (Fig 5E), congruent with the template which showed similar posterior movement (Fig 5F). In 2–3 hours old embryos, weak and uniform Bcd covered the anterior third of the embryo likely representing freely-moving Bcd, while the bulk of Bcd stayed localized to the anterior-most 20% of the embryo (Fig 5I), consistent with the template (Fig 5J). In late embryos (3–4 hours), Bcd showed further axial expansion of movement, including the bulk which also moved towards the interior (Fig 5M) consistent with the data of [8]. Concurrently, the Staufen protein has now migrated deeply into the interior of the embryo (Fig 5N), where it showed co-localization with the bulk of Bcd (Fig 5P). Another interesting feature was that Staufen disappeared rapidly at the posterior pole, while it persisted at the anterior pole (Fig 5B, 5F, 5J and 5N), suggesting distinct degradation mechanisms.

Our data on Bcd protein distribution in unfertilized embryos were in agreement with data of [8, 42]. However, our data on the behavior of Staufen was in true conflict with the interpretation of the SDD model in [42] which implied that the mRNA stayed anchored at the anterior tip at all times. We sought to resolve this apparent discrepancy and analyzed the *bcd* mRNA pattern in subsequent stages of unfertilized eggs. The fragile embryos were fixed well to survive the harsh treatment during *in situ* hybridization. As expected and in agreement with Fig 5B, the mRNA moved away from the tip during the early stages (Fig 5Q). More importantly, subsequent stages showed that the mRNA moved to the center of the embryo as a streak (Fig 5R and 5S, arrows) and not to the cortex, implying that the cortical network was not established. Furthermore, the streak mRNA did not seem to involve Staufen (compare Fig 5R and 5S with Fig 5F and 5J), suggesting that the *bcd* mRNA utilizes another adapter protein than Staufen for the transport along the streak. At late stages (3–4 hours), *bcd* mRNA encountered a barrier (Fig 5T, arrowhead) which prevented further transport towards the posterior. Our data demonstrates the presence of an internal MT network in unfertilized eggs, which serves as a path for migration of the mRNA, however, the cortical network is only built up upon fertilization [5]. Occasionally, in about 1–2% of fertilized wild-type embryos, a thin mRNA streak was observed, where the direction of the streak was always pointing towards the dividing nuclei (unpublished).

## Discussion

The present study sheds light onto the movement of the Bcd protein and demonstrates that the Bcd protein follows a discrete pathway at the cortex during early cleavage stages. The movement of Bcd during the early stages was never investigated experimentally until recently [8], but rather relied on assumptions based on the dogma of *bcd* gradient formation [1], the proposals of the SDD model, and on the simulation of protein movement by injecting dextrane particles [7].

Several assumptions were put forward in the past [6, 43–45] that should explain the apparent paradox of a diffusion coefficient of Bcd which was two orders of magnitude too low. Summarized in [44] were: 1) diffusion might be much higher in other compartments, for example within the center; 2) diffusion might be faster during nc 1–9, i. e. at a time when Bcd could not been observed by live imaging; 3) several fractions of Bcd molecules exist that have different diffusion properties; 4) active transport of Bcd.

For the first two assumptions, our data provides evidence that neither assumptions mentioned above can account for the diffusion properties as they were revealed. This for the following reasons: 1) the underlying cause for the protein gradient is the mRNA gradient [2, 3], 2) the protein moves along the cortex (this report).

### The yolk, a non-permissive-territory for Bcd movement

The cortical movement of Bcd in wild-type embryos revealed that the inner yolk mass was a non-permissive territory for Bcd movement (Fig 1). In the past, the yolk was considered to be a compartment where Bcd could diffuse fast enough to explain the apparent low diffusion constant [43]. Our data in Fig 1 refutes this possibility. In line with our observations for a distinct regionalization of Bcd movement, [46] described the inner yolk mass as an ellipsoid entity which morphologically behaved as a distinct unit during each syncytial nuclear cycle, while the cortex was described as a completely different entity.

Data about the content and structure of the inner yolk is sparse, mostly due to the inability of the laser of a confocal microscope to penetrate the dense yolk layer. Actin microfilaments in the deep yolk were described [47, 48], but require a more detailed description. As far as MTs are concerned, only the spindle apparatus during the nuclear cycles were bright enough to become visible [5].

Our drug treatment data demonstrated that the yolk became permissive for Bcd if embryos were treated with CC, vinblastine (Fig 3) or with phalloidin (Fig 4). The most pronounced of these cases was with vinblastine-treated embryos (Fig 3I–3K). The protein behaved exactly as the SDD model would have predicted, it moved to the posterior in a broad front (Fig 3K), while the mRNA remained at the tip.

In unfertilized eggs, the Bcd protein was also found in the yolk, but this observation was not the result of movement from the cortex, rather it represented *de novo* synthesis of Bcd from the template which was translocated to the yolk (Fig 5). During longer incubations, we observed some movement of the protein away from the source (Fig 5I and 5M), indicating that the yolk became “permissive” for Bcd movement under theses conditions. Possibly, in a unfertilized embryo, the components of the yolk alter with time, making it permissive for Bcd.

### The role of actin for Bcd stability and movement

In the past, it was shown that actin was instrumental in anchoring *bcd* mRNA to the anterior of stage 14 oocytes [17, 20], while only sparse information was available on actin’s role for the Bcd protein in the early embryo.

For the first time, we could demonstrate that actin has a profound impact on Bcd stability and movement in the embryo (Fig 4). Particular that actin-dependent, Bcd-stabilizing function (Fig 4A–4C), may constitute an undiscovered means to fine-tune Bcd gradient formation and nuclear filling, as actin is predominant at the cortex where gradient formation actually takes place. We noted that Bcd was particularly enriched around the energids under circumstances where actin was stabilized, i. e. upon phalloidin-treatment (Fig 4F and 4G). During MT-drug treatment, i. e. when actin function was not compromised, a similar enrichment of Bcd in energids was observed (Fig 3B, 3C, 3J and 3K). This observation suggests that Bcd tends to accumulate in territories where there is high levels of actin. Similar observations were made by [49]. Possibly, the energids could serve as a reservoir for Bcd to quicken the step of nuclear filling after the nuclear membrane has formed again.

In cases, where F-actin was destroyed (Fig 4A–4C, S3C and S3D Fig), we also noted a second function of F-actin: aiding Bcd movement to the posterior. While degradation could contribute to a general picture that there was less posterior migration due to a lack of signal, increase of the gain during the confocal analysis confirmed that Bcd movement is indeed impeded (not shown). While actin-dependent long-range movement of Bcd is an unlikely process to occur in wild-type embryos, it suggests that actin may play a role for short range movement of Bcd, e. g. from the site of synthesis to the energids or directly to the nuclei.

### Interior transport of *bcd* mRNA

In unfertilized eggs, the cortical MT network for *bcd* mRNA transport was not activated [5] and hence the *bcd* mRNA was not transported along the cortex (Fig 5). Instead, the mRNA was transported, presumably via MTs in the yolk into the middle of the embryo. Concomitantly, the Bcd protein was translated and was confined to the interior of the egg (Fig 5A, 5E, 5I and 5M). Although we could confirm the data of [42], i. e. that a longer, shallower Bcd gradient was observed compared to fertilized embryos of the same age, we could not support their claim that the SDD model was the basis for their observation. Our results demonstrated active transport of the mRNA into the interior of the egg, followed by translation [1]. The weakness of the conclusions drawn by [42] was that they were based on the assumption that the mRNA would stay anchored at the tip which was not the case (Fig 5Q–5T). Our observations were not consistent with an involvement of the SDD model to explain the apparent extended range of Bcd diffusion. Another striking observation was that the inward migration of the mRNA in unfertilized embryos was faster than cortical transport in fertilized embryos (Fig 5R and 5S), raising the possibility that 1) either the tubulin composition of the two MT networks was different from that at the cortex; 2) the interior MT network was oriented more parallel to the A-P axis than the cortical one; 3) the internal MT persisted during all nuclear cycles.

## Conclusions

Why does localization and movement of the Bcd protein at the cortex make sense? In the past, we and others have shown that the mRNA gradient follows a similar discrete cortical path as the protein [2, 3, 5, 8]. Hence, the protein as a direct consequence of its template is located in close proximity to the mRNA. Since the egg undergoes strong periplasmic contractions during each nuclear cycle [46], any protein gradient generated by free movement from the tip would suffer from unwanted turbulence. However, since the information of the gradient is already stored at the level of the mRNA [2, 3, 5], the mRNA associated with microtubules is much more resistant against cytoplasmic turbulences than the freely floating Bcd protein. Another feature is that local Bcd synthesis and the shuffling activity of Bcd between the periplasm and the nucleus might be under intricate control. Reported as a rather intriguing result, the concentration of Bcd in nuclei after every mitosis is surprisingly constant, taking into account that number and volume of nuclei vary with each nuclear cycle [43]. Here, we wish to add a rather simple explanation for this seemingly intriguing result: Since each point along the A-P axis experiences an increase of *bcd* mRNA concentration during each nuclear cycle, the number of templates for the translation of Bcd are adjusted for the need of each nuclear cycle. This model could be extended by proposing that during each nuclear cycle, the Bcd protein could be degraded, facilitated by the presence of the PEST domain [45, 50, 51]. This model has barely been taken into consideration in the past, except in [43]. *De novo* synthesis of Bcd takes maximally 2 minutes [3, 37], permitting enough time to fill the nuclei with the correct amount of Bcd, based on the mRNA template. Moreover, since Bcd is translated locally, we envision that there might be tightly-adjusted control mechanisms linking the process of translation to nuclear translocation, thereby allowing to adjust intricately the concentration of Bcd within the nuclei.

## Supporting information

S1 Fig Comparison of Bcd patterns of *bcd*^*+5+8*^ and wild-type embryos. (A), (D), (G) nc 6 *bcd*^*+5+8*^ embryo stained for Bcd (A) using saturation of intensities at the tip, color conversion (D) from 0–255 (8 bit), and Staufen (G). (B), (E), (H) nc 6 wild-type embryo stained for Bcd (B) and analyzed using identical confocal parameters as for (A), color conversion (E) with a maximum scale of 1/3 of (D), i. e. from 0–85, and Staufen (G). (C), (F), (I) identical nc 6 wild-type embryo as in (B), recorded using confocal parameters for saturation of intensities at the tip, color conversion (F) with maximum scale of 255, and DAPI to reveal nuclei (I). Note that *bcd*^*+5+8*^ embryos express about 3 times more Bcd than wild-type embryos, best seen at color conversion between (D) and (E). Likewise, Staufen as a read-out for *bcd* mRNA is equally stronger in *bcd*^*+5+8*^ embryos (G), compared to wild-type embryos (H).
(TIF)

S2 Fig Movement of small molecules into *Drosophila* embryos. (A-D) nc 8 embryo exposed for 2 hours to hypoxia, simultaneously incubated with 3 molecular markers and recorded as a single stack in different channels, revealing Hoechst 33342 (A), Sytox Green (B), TO-PRO3 (C) and merge of (A-C) in (D). (E-H), high magnification of a single stack recording of the embryo in (A-D) showing Hoechst 33342 (E), Sytox Green (F), TO-PRO3 (G) and merge of (E-F) in (H). Note the condensation of the chromatin under hypoxic conditions at the inner surface of nuclei, first described by [9].
(TIF)

S3 Fig Effect of phalloidin and latrunculin B on cortical actin microfilaments. (A-D) nc 6 embryos exposed for 2 hours to hypoxia and to phalloidin (A, B) and to latrunculin B (C, D). (A) sagittal confocal section, actin staining as revealed with mab JLA20 (red) together with DAPI (blue). (B) cortical confocal section of the anterior tip at high magnification of the same embryo as in (A), stained for actin (red). (C) sagittal confocal section, actin staining as revealed with mab JLA20 (red), together with DAPI (blue). (D) cortical confocal section of the anterior tip at high magnification of the same embryo as in (C), stained for actin (red). Note the extended actin microfilaments upon phalloidin-treatment (B), compared to the globular actin appearance upon latrunculin B-treatment (D).
(TIF)

S1 Table *bcd*^*+5+8*^ phenotypes. Percentages of cuticular phenotypes of 3 h hypoxic and 36 h recovered embryos (left) and *bcd*^*+5+8*^ embryos (right). 3 classes were compared, normal cuticle (blue), mild cuticle phenotype (red) and severe cuticle phenotype (green).
(TIF)
