# Correction: Cortical movement of Bicoid in early *Drosophila* embryos is actin- and microtubule-dependent and disagrees with the SDD diffusion model

**DOI:** 10.1371/journal.pone.0196144

**Published:** 2018-04-18

**Authors:** Xiaoli Cai, Mira Akber, Alexander Spirov, Stefan Baumgartner

S1 Table is incorrect. Please see the correct S1 Table below.

## Supporting information

S1 Table *bcd*^+5+8^ phenotypes. Percentages of cuticular phenotypes of 3 h hypoxic and 36 h recovered embryos (left) and *bcd*^+5+8^ embryos (right). 3 classes were compared, normal cuticle (blue), mild cuticle phenotype (red) and severe cuticle phenotype (green).
(TIF)

## References

[1] Cai X, Akber M, Spirov A, Baumgartner S (2017) Cortical movement of Bicoid in early *Drosophila* embryos is actin- and microtubule-dependent and disagrees with the SDD diffusion model. PLoS ONE 12(10): e0185443 https://doi.org/10.1371/journal.pone.0185443 28973031 10.1371/journal.pone.0185443 PMC5626467

