# Supplementary figures and images for: Cortical movement of Bicoid in early Drosophila embryos is actin- and microtubule-dependent and disagrees with the SDD diffusion model
Source: PLoS One. 2017 Oct 3;12(10):e0185443. doi: 10.1371/journal.pone.0185443 (PMC5626467; doi:10.1371/journal.pone.0185443)

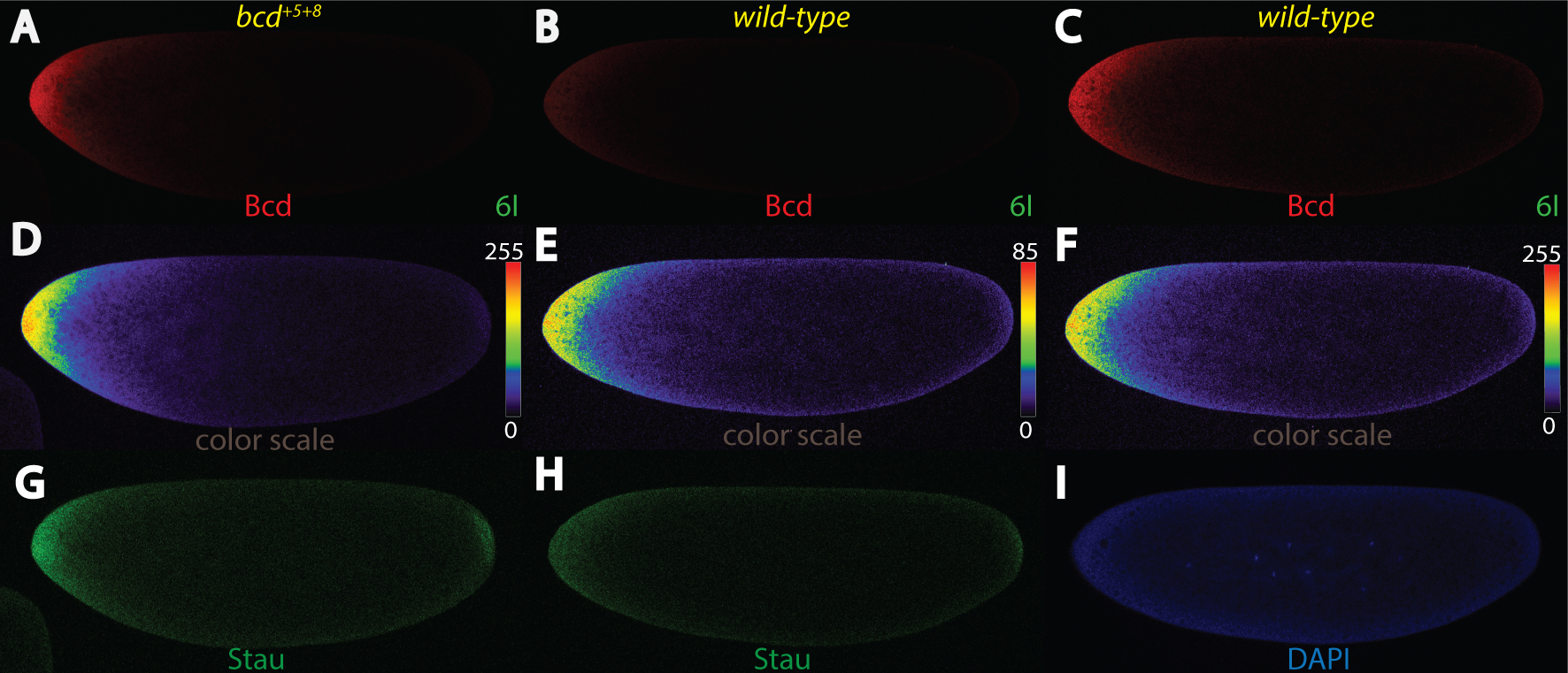

Supplement: S1 Fig — (A), (D), (G) nc 6 bcd+5+8 embryo stained for Bcd (A) using saturation of intensities at the tip, color conversion (D) from 0–255 (8 bit), and Staufen (G). (B), (E), (H) nc 6 wild-type embryo stained for Bcd (B) and analyzed using identical confocal parameters as for (A), color conversion (E) with a maximum scale of 1/3 of (D), i. e. from 0–85, and Staufen (G). (C), (F), (I) identical nc 6 wild-type embryo as in (B), recorded using confocal parameters for saturation of intensities at the tip, color conversion (F) with maximum scale of 255, and DAPI to reveal nuclei (I). Note that bcd+5+8 embryos express about 3 times more Bcd than wild-type embryos, best seen at color conversion between (D) and (E). Likewise, Staufen as a read-out for bcd mRNA is equally stronger in bcd+5+8 embryos (G), compared to wild-type embryos (H). (TIF) [file pone.0185443.s001.tif]

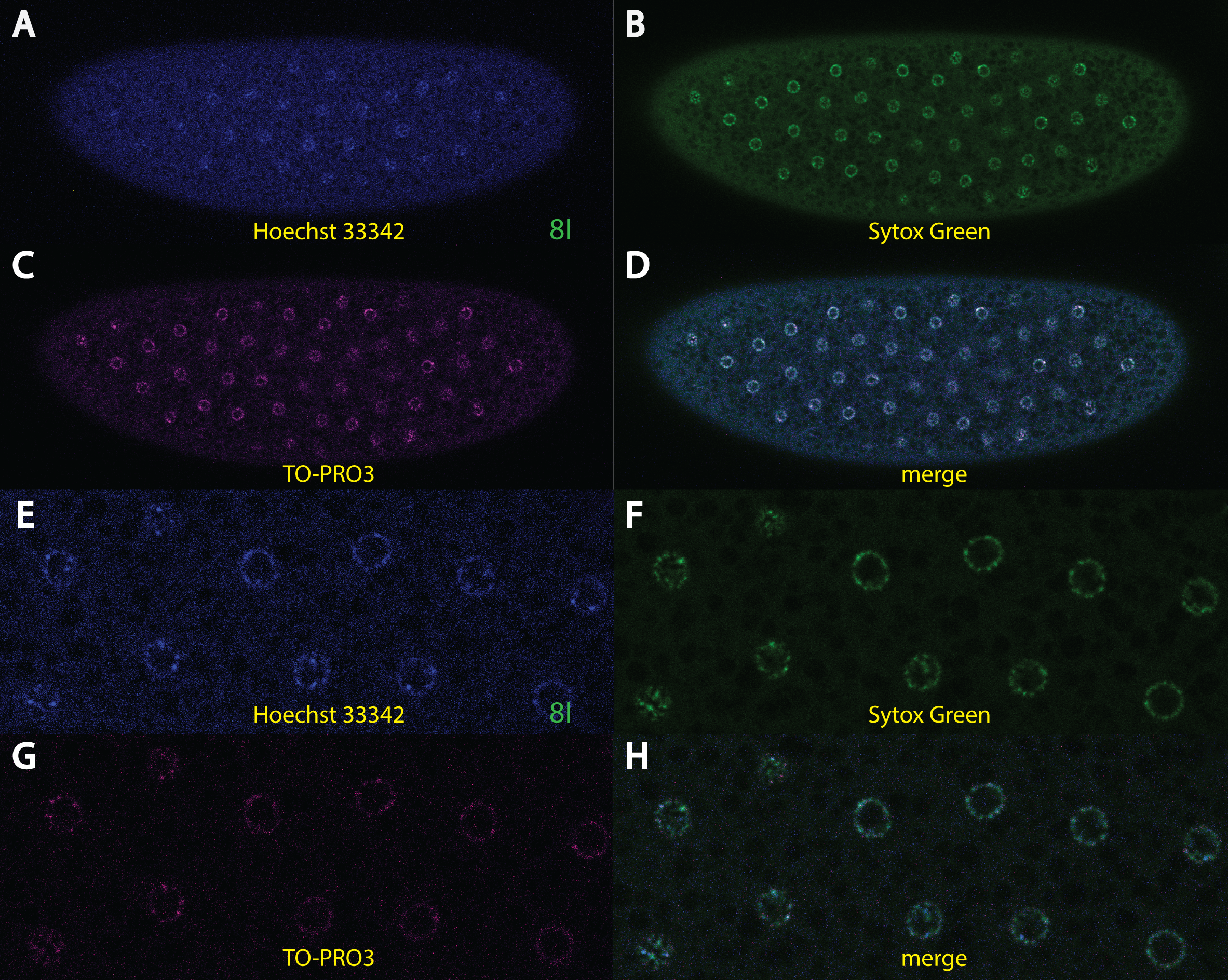

Supplement: S2 Fig — (A-D) nc 8 embryo exposed for 2 hours to hypoxia, simultaneously incubated with 3 molecular markers and recorded as a single stack in different channels, revealing Hoechst 33342 (A), Sytox Green (B), TO-PRO3 (C) and merge of (A-C) in (D). (E-H), high magnification of a single stack recording of the embryo in (A-D) showing Hoechst 33342 (E), Sytox Green (F), TO-PRO3 (G) and merge of (E-F) in (H). Note the condensation of the chromatin under hypoxic conditions at the inner surface of nuclei, first described by [9]. (TIF) [file pone.0185443.s002.tif]

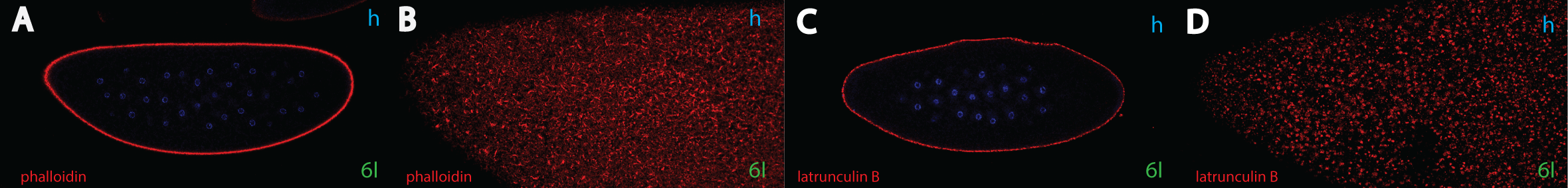

Supplement: S3 Fig — (A-D) nc 6 embryos exposed for 2 hours to hypoxia and to phalloidin (A, B) and to latrunculin B (C, D). (A) sagittal confocal section, actin staining as revealed with mab JLA20 (red) together with DAPI (blue). (B) cortical confocal section of the anterior tip at high magnification of the same embryo as in (A), stained for actin (red). (C) sagittal confocal section, actin staining as revealed with mab JLA20 (red), together with DAPI (blue). (D) cortical confocal section of the anterior tip at high magnification of the same embryo as in (C), stained for actin (red). Note the extended actin microfilaments upon phalloidin-treatment (B), compared to the globular actin appearance upon latrunculin B-treatment (D). (TIF) [file pone.0185443.s003.tif]

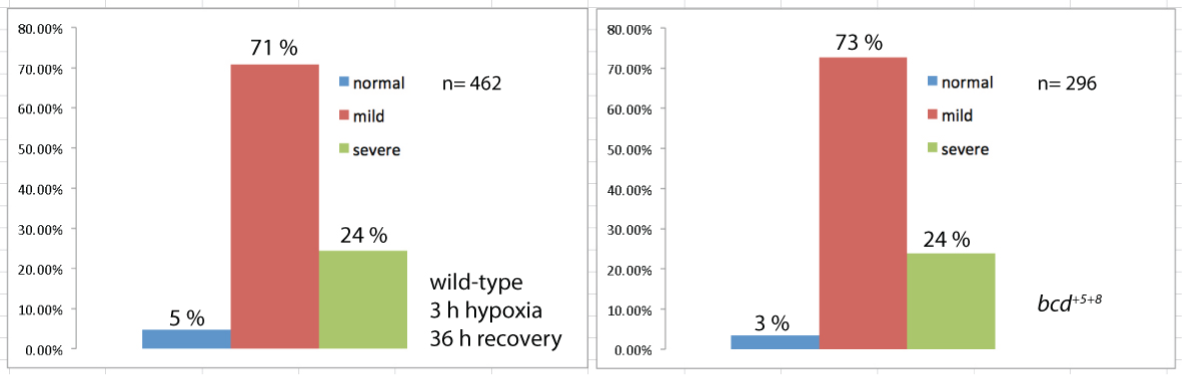

Supplement: S1 Table — Percentages of cuticular phenotypes of 3 h hypoxic and 36 h recovered embryos (left) and bcd+5+8 embryos (right). 3 classes were compared, normal cuticle (blue), mild cuticle phenotype (red) and severe cuticle phenotype (green). (TIF) [file pone.0185443.s004.tif]
