# Supplementary figures and images for: Correction: Cortical movement of Bicoid in early Drosophila embryos is actin- and microtubule-dependent and disagrees with the SDD diffusion model
Source: PLoS One. 2018 Apr 18;13(4):e0196144. doi: 10.1371/journal.pone.0196144 (PMC5906000; doi:10.1371/journal.pone.0196144)

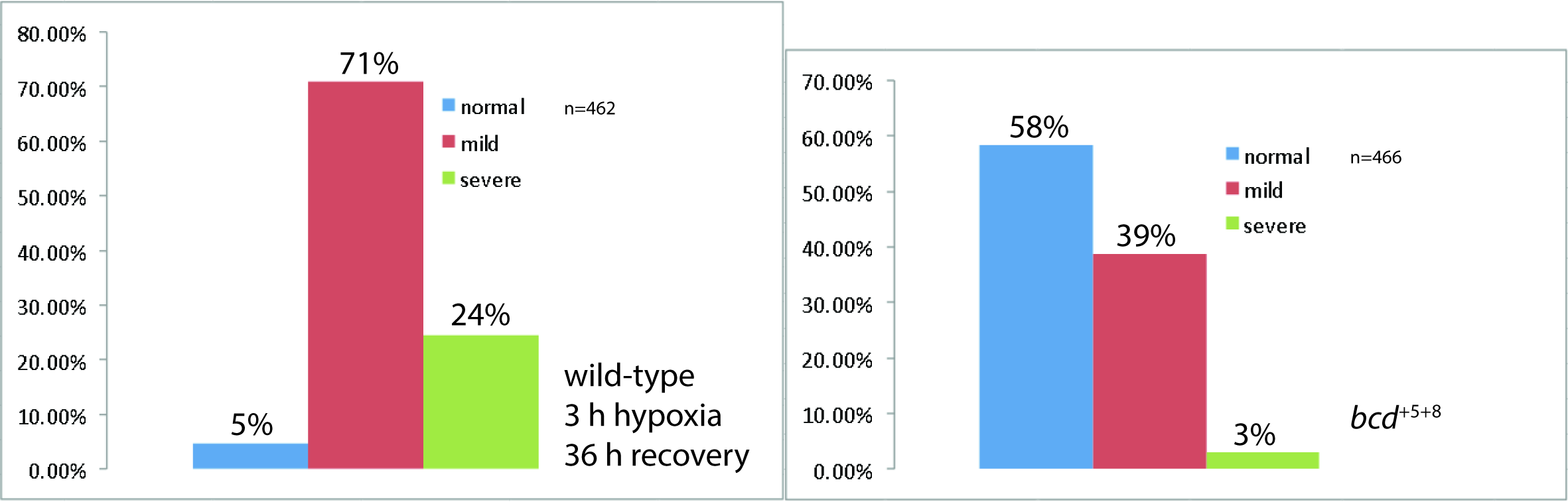

Supplement: S1 Table — Percentages of cuticular phenotypes of 3 h hypoxic and 36 h recovered embryos (left) and bcd+5+8 embryos (right). 3 classes were compared, normal cuticle (blue), mild cuticle phenotype (red) and severe cuticle phenotype (green). (TIF) [file pone.0196144.s001.tif]
